# Multi‐Mechanistic Effects of *Xylopia aethiopica* Fruits on Uterine Fibroids: Insights From In Vivo and In Silico Evidence

**DOI:** 10.1002/cbdv.71704

**Published:** 2026-09-15

**Authors:** Adedokun Oluwasegun, Ume Ogochukwu, Akinsola Olubiyi, Agada Emmanuel, Adebiyi Omowumi, Idogho Eseoghena, Adedokun Folake, Adekunle Temilade

**Affiliations:** ^1^ CBIOS – Research Center for Bioscience and Health Technologies Universidade Lusofona Lisbon Portugal; ^2^ Department of Pharmaceutical Chemistry Igbinedion University Okada Nigeria; ^3^ Cancer and Reproductive Research Group College of Pharmacy Igbinedion University Okada Nigeria; ^4^ Department of Physiology (Pharmacology unit) Faculty of Medical Sciences Redeemer's University Ede Nigeria

**Keywords:** estradiol, molecular docking, multi‐target therapy, progesterone, uterine fibroids, *Xylopia aethiopica* fruits

## Abstract

Uterine fibroids (leiomyomas) are hormone‐dependent benign tumors driven by progesterone and estradiol. This study investigated the multi‐mechanistic effects of *Xylopia aethiopica* on fibroid pathophysiology using in vivo and in silico approaches. Thirty Wistar rats were induced with fibroids via monosodium glutamate (MSG) and treated with varying doses (100, 200, 400 mg/kg) of *X. aethiopica* extract. Serum progesterone and estradiol were measured, and molecular docking and protein–protein interaction analyses assessed potential mechanisms. MSG significantly elevated progesterone (30.12 ± 2.43 ng/mL) and estradiol (48.56 ± 3.24 pg/mL), while *X. aethiopica* normalized these hormones in a dose‐dependent manner. Docking studies revealed strong interactions of bioactive compounds, including aporphine, xylopioxyde, and liriodenine with progesterone, estrogen, VEGFR2, and TGF‐β receptors, suggesting hormonal, anti‐angiogenic, and anti‐fibrotic effects. These findings indicate that *X. aethiopica* exerts pleiotropic actions to restore endocrine balance and inhibit fibroid‐promoting pathways, supporting its potential as a natural, multi‐targeted therapeutic agent for uterine fibroids.

## Graphical abstract

*Xylopia aethiopica* fruit extract modulates progesterone and estradiol levels in monosodium glutamate‐induced uterine fibroid models. In silico analyses reveal strong binding of its phytochemicals to progesterone, estrogen, VEGFR2, and TGF‐β receptors, indicating multi‐target anti‐fibroid actions involving hormonal regulation, anti‐angiogenesis, and anti‐fibrotic pathways in uterine fibroid pathophysiology.

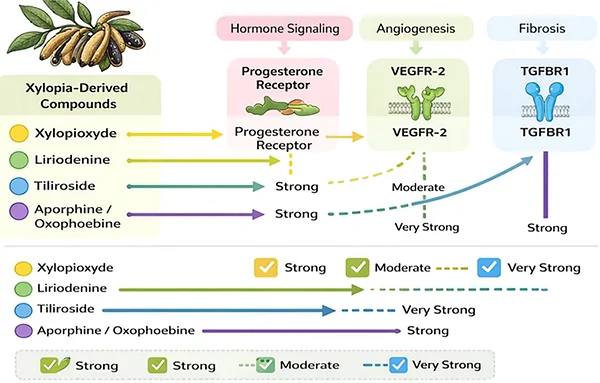

**Abbreviations**
42Q: native ligand co‐crystallized with VEGFR2 (3VHE)
Aporphine: alkaloid compound isolated from *Xylopia aethiopica*
ECM: extracellular matrix
EIA: enzyme immunoassay
ESR1: estrogen receptor alpha
EST: native ligand co‐crystallized with estrogen receptor alpha (5GS4)
ETC: native ligand co‐crystallized with estrogen receptor beta (1L2J)
FGF2: fibroblast growth factor 2
GLY: glycine (amino acid residue)
HIS: histidine (amino acid residue)
HSP90AA1/HSP90AB1: heat shock protein 90 alpha/beta family members
ILE: isoleucine (amino acid residue)
KDR: kinase insert domain receptor (VEGFR2)
LEU: leucine (amino acid residue)
Liriodenine: alkaloid compound isolated from *Xylopia aethiopica*
LSD: least significant difference (post hoc statistical test)
MET: methionine (amino acid residue)
MSG: monosodium glutamate
NCOR1/NCOR2: nuclear receptor corepressor 1/2
NCOA1/NCOA3: nuclear receptor coactivator 1/3
NRP1: neuropilin‐1
Oxophoebine: alkaloid compound isolated from *Xylopia aethiopica*
PDGFC: platelet‐derived growth factor C
PBS: phosphate‐buffered saline
PECAM1: platelet endothelial cell adhesion molecule 1
PGF: placental growth factor
PGR: progesterone receptor
π–π/π–σ/π–alkyl: types of π interactions (aromatic/non‐covalent interactions in docking studies)
RMSD: root mean square deviation
SMAD2/3: mothers against decapentaplegic homolog 2/3
SMAD6/7: inhibitory SMAD proteins
SMURF2: SMAD‐specific E3 ubiquitin protein ligase 2
SRC: proto‐oncogene tyrosine‐protein kinase Src
TGF‐β1 (TGFB1): transforming growth factor beta 1
TGFBR1: transforming growth factor beta receptor type 1
TGFBR2: transforming growth factor beta receptor type 2
Tiriloside: bioactive compound isolated from *Xylopia aethiopica*
VAL: valine (amino acid residue)
VEGFB/VEGFC/VEGFD: vascular endothelial growth factor B/C/D
VEGFR2: vascular endothelial growth factor receptor 2
Xylopioxyde: bioactive diterpene isolated from *Xylopia aethiopica*
YAP1: yes‐associated protein 1
NEDD4L: neural precursor cell expressed developmentally down‐regulated protein 4‐like

## 1 Introduction

Leiomyomas or myomas are other names for uterine fibroids, which are the most prevalent type of benign uterine tumors. Since they are monoclonal tumors of the smooth muscle of the uterus, the myometrium is where they originate [1]. 50%–60% of women have leiomyoma, which rise to 70% by the age of 50. In 30% of instances, these tumors result in morbidity because of aberrant pelvic pressure (constipation, tenesmus, and urine symptoms) and uterine bleeding (heavy menstrual bleeding that causes anaemia). Infertility, obstetric problems, pelvic masses, and pelvic pain are among the clinical manifestations of uterine leiomyoma [2]. Uterine fibroids can induce a variety of severe and persistent symptoms, or they can be asymptomatic. Fatigue, painful periods, and excessive menstrual bleeding—which can result in anaemia—are the most frequent presenting symptoms [3]. Other symptoms of uterine fibroids include abdominal protuberance, painful bowel movements or pelvic pressure, noncyclic discomfort, and bladder or bowel malfunction that causes pain, constipation, or urine incontinence or retention [4]. Additionally, 4–10 uterine fibroids may be linked to reproductive issues such as decreased fertility, pregnancy loss and difficulties, and unfavorable obstetric outcomes [5].

In Africa, the reported prevalence of uterine fibroids varies from 3% to 70%, contingent on study methodology and diagnostic standards [6]. In Nigeria, the lifetime incidence is roughly 70%–80% by the age of 50, with a frequency of 12%–30% among women of reproductive age [7]. The greatest incidence is seen in the 26–45 age range, with nulliparous and overweight people having greater rates. Uterine fibroids pose a significant threat to reproductive health in African communities [8]. The management of uterine fibroids involves a multidisciplinary approach, ranging from medical therapy and minimally invasive procedures to definitive surgical interventions, depending on the size of the fibroid, symptom severity, and the patient's reproductive goals [9]. Conventional treatments for uterine fibroids, including medical therapy and surgical interventions, have several important limitations. Medical treatments such as hormonal agents and gonadotropin‐releasing hormone analogues primarily provide temporary relief of symptoms rather than permanent resolution, as fibroids often regrow once therapy is discontinued [10]. Their long‐term use is limited by significant side effects, including menopausal symptoms, bone loss, and hormonal disturbances, and they may not be suitable for women seeking to conceive. In addition, most medications have limited ability to substantially reduce fibroid size, particularly in cases of large or multiple lesions [10].

Surgical approaches, while more definitive, are associated with their own challenges. Myomectomy carries a substantial risk of fibroid recurrence, whereas hysterectomy results in permanent loss of fertility and may not be acceptable to many women. Both procedures are invasive, costly, and associated with operative risks, prolonged recovery periods, and potential postoperative complications. Minimally invasive procedures, such as uterine artery embolization and focused ultrasound therapy, are not universally applicable, have limited availability, and raise concerns regarding long‐term fertility and pregnancy outcomes [11]. Collectively, these limitations underscore that conventional fibroid treatments are often symptom‐oriented, may compromise reproductive potential, and can impose significant physical, psychological, and socioeconomic burdens on affected women [12].

Several medicinal plants are traditionally used across Africa for the management of uterine fibroids, largely due to their perceived hormonal, anti‐inflammatory, and antiproliferative effects. Commonly cited plants include *Vitex doniana*, *Newbouldia laevis*, *Alchornea cordifolia*, *Curcuma longa* (turmeric), *Azadirachta indica* (neem), and so forth [13]. *Xylopia aethiopica* was selected for this research because of its widespread ethnomedicinal use in West and Central Africa for female reproductive disorders and its rich phytochemical profile, particularly diterpenoids and other bioactive compounds with reported anti‐inflammatory, antioxidant, and antiproliferative properties [13]. Emerging experimental evidence suggests that these compounds may influence estrogen‐related pathways and inhibit smooth muscle cell proliferation, key mechanisms involved in fibroid development. In addition, the plant's availability, long history of traditional use, and relatively limited scientific exploration in the context of uterine fibroids make *X. aethiopica* a strong and rational candidate for further investigation [13]. The aim of this research is to scientifically evaluate the therapeutic potential of *X. aethiopica* in the management of uterine fibroids, with particular emphasis on its bioactive compounds and their mechanisms of action.

## 2 Materials and Methods

### 2.1 Chemical

Artificial monosodium glutamate (MSG) was purchased from a vendor store in Mushin, Lagos, Nigeria.

### 2.2 Plant Material

Fruits of *X. aethiopica* (Dunal) A. Rich. were collected in Okada, Edo State, Nigeria, by Dr. Ume Ogochukwu of the College of Pharmacy, Igbinedion University, Okada, Nigeria. The plant material was identified and authenticated by Dr. Gabriel Benjamin of the Department of Plant Biology and Biotechnology, University of Benin, Benin City, Nigeria. A voucher specimen was deposited at the Herbarium Section of the University of Benin, Benin City, Nigeria, under voucher number UBH‐R633. The plant sample was air‐dried in the laboratory for 5 days at room temperature followed by oven drying at 40°C. Grinding to powder form was carried out using a laboratory electric mill. The powdered sample was kept in an airtight container until required.

### 2.3 Extraction

Extraction of 1.2 kg of the powdered leaves of *X. aethiopica* was done with absolute methanol using Soxhlet extraction, followed by concentration using a rotary evaporator and the extract obtained was weighed and kept in the refrigerator 4°C.

### 2.4 Experimental Animals

Thirty wistar rats weighing 200–230 g were obtained from disease‐free stocks maintained in the central animal house of the Igbinedion University, Nigeria. The animals were randomly assigned into five study groups on the basis of average body weight and litter origin. Each rat in a study group was individually housed in a stainless cage with plastic bottom grid and a wire screen top. The animal's room was adequately ventilated and kept at a room temperature and relatively humidity of 29 ±2 C and 40%–70%, respectively, with a 12 h natural light‐dark cycle. Animals were fed *ad libitum* with water and rats chew (Live stock feeds Ltd, Calabar, Nigeria). Good hygiene was maintained by constant cleaning (removal of feces and spilled water from cages daily). All animal experimental procedures were conducted in accordance with the guidelines for the care and use of laboratory animals. Ethical approval for the study was obtained from the Faculty of Life Sciences Ethical Committee, University of Benin, Benin City, Nigeria, under approval number LS171186. All efforts were made to minimize animal suffering and to reduce the number of animals used in the study.

Group A (Negative control) received 200 mg/kg of MSG only.

Group B was administered with 100 mg/kg of *X. aethiopica* + 200 mg/kg of MSG only.

Group C received 200 mg/kg body weight of *X. aethiopica* and 200 mg/kg of MSG.

Group D was given 400 mg/kg body weight of *X. aethiopica* and 200 mg/kg of MSG.

Group E (Normal control) received 1 mL/kg of distilled water [14].

#### 2.4.1 Sample Preparation

All rats were fasted overnight and, on day 28, were anesthetized and euthanized by cervical dislocation. Blood was collected into EDTA‐sterilized sample bottles and plain bottles after the treatment was terminated. The uterus were taken from the animal and weighed after being blotted dry with filter paper.

#### 2.4.2 Determination of Oestrogen Level

This determination followed a previously reported procedure which described an enzyme immunoassay (EIA) used to determine oestrogen levels in the bloodstream. Using acetic acid to adjust the pH of the serum sample (4 mL), it was extracted with 12 mL of diethyl ether (pH 3.5), which was then vaporized and re‐extracted with diethyl ether (pH 3.5). Following evaporation, the extract was dissolved in 12 mL of assay buffer (40 mM PBS, 0.1% BSA, pH 7.2) and pooled to yield 3.2 mL in PBS (pH 7.5); the sample was then dissolved in 12 mL of 100% methanol to obtain the final concentration. The amount of estrogen found in each serum sample (4 mL) was determined. In addition to the discovery and documentation of the retention time (11.4 min) and the antigen–antibody reaction, from 0.15 pg (80% labeled antigen) to 7.2 pg (80% displacement of labeled antigen), the working interval was measured (20% displacement of the labeled antigen of oestrogen per 4 mL). The calibration curve for the EIA was done with 40% methanol [15].

### 2.5 Probable Mechanism Determination Using In Silico Model

#### 2.5.1 Selection and Preparation of Ligands

Bioactive compounds isolated from *X. aethiopica* were selected based on documented phytochemical and pharmacological reports. The chemical structures of the isolated compounds were obtained from literature sources and manually sketched using chemical structure drawing software. The two‐dimensional (2D) structures were converted into 3D conformations and subjected to energy minimization using an appropriate molecular mechanics force field to remove steric clashes and obtain stable geometries. All ligand structures were protonated appropriately, explicit hydrogen atoms were added, and partial atomic charges were assigned. Rotatable bonds were defined to allow conformational flexibility during docking. The optimized ligands were saved in docking‐compatible file formats for subsequent molecular docking analysis [16].

#### 2.5.2 Retrieval and Preparation of Target Proteins

The 3D crystal structures of the target proteins were retrieved from the Protein Data Bank (PDB) [17]. The selected proteins, their PDB identification codes, and biological relevance are as follows:

*2W8Y*: RU486 bound to the progesterone receptor in a destabilized agonistic conformation.

*3VHE*: Crystal structure of human VEGFR2 kinase domain with a novel pyrrolopyrimidine inhibitor.

*5E90*: TGF‐BETA receptor type 1 kinase domain in complex with 3‐amino‐6‐[4‐(2‐hydroxyethyl)phenyl]‐n‐[4‐(morpholin‐4‐yl)pyridin‐3‐yl]pyrazine‐2‐carboxamide.

*5GS4*: Crystal structure of estrogen receptor alpha (ESR1) in complex with a stabilized peptide antagonist.

*1L2J*: Human estrogen receptor beta ligand‐binding domain in complex with (R,R)‐5,11‐cis‐diethyl‐5,6,11,12‐tetrahydrochrysene‐2,8‐diol.

These proteins were selected due to their established or reported roles in uterine contraction, smooth muscle regulation, prostaglandin signaling, hormonal modulation, and calcium‐mediated pathways, which are central to uterotonic activity. Protein preparation involved removal of co‐crystallized ligands, ions, and water molecules to avoid interference with ligand binding, except for structurally essential residues. Polar hydrogen atoms were added, appropriate charges were assigned, and missing residues were corrected where necessary. The prepared protein structures were energy‐optimized and saved in suitable formats for docking. Binding sites were identified based on the coordinates of co‐crystallized ligands present in the protein structures where available. For proteins lacking bound ligands, active sites were determined using reported functional residues from literature and structural cavity prediction tools. Grid boxes were defined to encompass the active site residues adequately, ensuring sufficient space for ligand flexibility and optimal docking accuracy [18].

#### 2.5.3 Molecular Docking Procedure

Molecular docking simulations were carried out to investigate the binding affinity and interaction patterns of *X. aethiopica*‐derived compounds with the selected protein targets. Docking was performed using a validated molecular docking algorithm employing a stochastic search method to explore ligand conformations and orientations within the binding pocket [19].

For each ligand–protein pair, multiple docking runs were conducted to ensure reproducibility. The docking protocol generated several binding poses, and the best pose was selected based on the lowest binding free energy and favorable interaction geometry. Binding affinities were expressed in kcal/mol, with more negative values indicating stronger predicted binding. To validate the docking protocol, re‐docking of the native co‐crystallized ligand was performed for applicable protein targets. The docked pose was superimposed on the crystallographic pose, and the root mean square deviation (RMSD) was calculated. An RMSD value of ≤ 2.0 Å was considered indicative of a reliable docking protocol [19].

#### 2.5.4 Protein–Ligand Interaction Analysis

The docked protein–ligand complexes were analyzed to identify key intermolecular interactions, including hydrogen bonding, hydrophobic interactions, π–π stacking, van der Waals forces, and electrostatic interactions. Visualization and interaction profiling were performed using molecular visualization software. Amino acid residues contributing to binding were identified and compared across all target proteins to elucidate shared and distinct interaction patterns. Docking scores and interaction profiles were systematically compared across all ligands and protein targets. Compounds exhibiting strong binding affinity, stable interactions with critical active‐site residues, and consistent multi‐target engagement were identified as potential lead compounds. These findings provide computational support for the uterotonic potential of *X. aethiopica*‐derived compounds and form a basis for further experimental validation [20].

### 2.6 Statistical Analysis

The data were analyzed using Microsoft Excel and Graph pad prism 7. Results were presented as (mean ± SEM). The parameters for all groups were compared using analyses of variance (ANOVA). Post hoc analysis was done using least square difference (LSD). Differences in means were considered significant at values of *p ≤ *0.05.

## 3 Results and Discussion

### 3.1 Effect of *X. aethiopica* on Serum Progesterone

**FIGURE 1:**
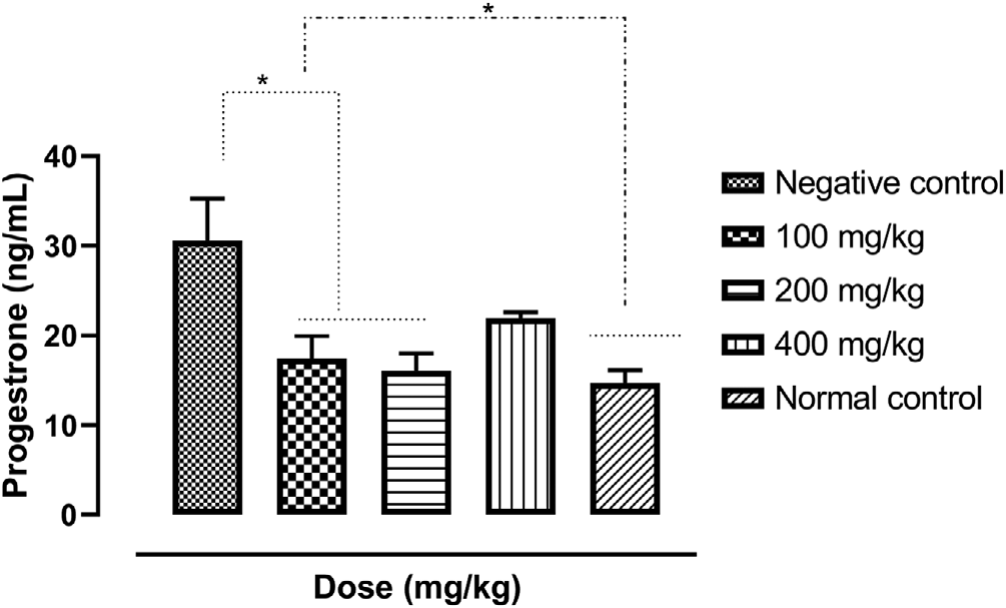
Influence of plant extract on serum progesterone level in MSG‐induced fibroid animals. The values above are mean of six replicates (*n* = 6). Values with superscripts * indicate significant difference relative value of negative control *p* ≤ 0.05.

The effect of *X. aethiopica* extract on serum progesterone concentration in MSG‐induced fibroid rats is presented in Figure 1. Administration of MSG alone (Group A) produced a marked and statistically significant (*p* ≤ 0.05) elevation in serum progesterone level (30.12 ± 2.43 ng/mL) compared with the normal control group (Group E), which recorded the lowest mean value (14.35 ± 1.98 ng/mL). Co‐administration of *X. aethiopica* extract with MSG resulted in a dose‐dependent reduction in progesterone concentration relative to the MSG‐treated group (Figure 1).

Specifically, animals treated with 100 mg/kg of *X. aethiopica* (Group B) showed a progesterone level of 16.24 ± 1.31 ng/mL, while the 200 mg/kg dose group (Group C) recorded 21.02 ± 1.87 ng/mL. The highest dose of 400 mg/kg (Group D) produced a level of 15.36 ± 1.22 ng/mL. Statistical analysis revealed that all extract‐treated groups had significantly lower progesterone concentrations than the MSG‐only group, whereas no significant difference was observed between the normal control and the 400 mg/kg group (*p* *≤* 0.05).

However, it is noteworthy to state that the administration of *X. aethiopica* extract significantly modulates serum progesterone levels in MSG‐induced fibroid rats. In the MSG‐only group (Group A), a marked elevation in serum progesterone (30.12 ± 2.43 ng/mL) was observed compared with the normal control (14.35 ± 1.98 ng/mL), confirming that MSG exposure disrupts endocrine function and promotes hyperprogesteronemia (Figure 1).

This finding aligns with previous reports showing that MSG can alter hypothalamic–pituitary–ovarian (HPO) axis function, leading to hormonal imbalance and enhanced steroidogenesis in animal models [21, 22]. Elevated progesterone levels are particularly relevant in fibroid pathophysiology, as progesterone is known to promote fibroid growth through stimulation of cell proliferation and extracellular matrix (ECM) deposition [23]. Co‐administration of *X. aethiopica* extract resulted in a dose‐dependent normalization of serum progesterone, with the 100 and 400 mg/kg doses reducing progesterone levels to near‐normal values (16.24 ± 1.31 and 15.36 ± 1.22 ng/mL, respectively). This effect indicates that *X. aethiopica* possesses bioactive compounds capable of modulating steroidogenesis, possibly through interaction with ovarian steroidogenic enzymes or by influencing hypothalamic–pituitary signaling. Previous studies have reported that *X. aethiopica* contains alkaloids, flavonoids, and polyphenols, which exhibit antioxidant, anti‐inflammatory, and hormonal modulatory effects [24, 25]. Such phytochemicals could mitigate MSG‐induced oxidative stress in ovarian tissue, thereby restoring normal progesterone synthesis. Interestingly, the 200 mg/kg dose (Group C) produced a slightly higher progesterone level (21.02 ± 1.87 ng/mL) compared to the lower and higher doses, suggesting a potential nonlinear dose‐response, which may be due to pharmacokinetic or receptor‐mediated effects. Similar biphasic responses have been reported with plant extracts affecting reproductive hormones, highlighting the complexity of plant–hormone interactions [26]. Overall, these results indicate that *X. aethiopica* has a protective and regulatory effect on serum progesterone in fibroid models. By attenuating MSG‐induced hyperprogesteronemia, the extract may contribute to preventing fibroid progression and maintaining reproductive hormonal balance. These findings corroborate earlier reports on the therapeutic potential of *X. aethiopica* and other medicinal plants in managing gynecological disorders linked to hormonal imbalance [27].

It is noteworthy to state that the MSG‐induced model was employed in this study based on evidence that chronic MSG exposure disrupts hypothalamic neuronal function and alters HPO axis regulation, leading to elevated circulating sex steroid hormones and reproductive tissue dysfunction. Although this model does not reproduce histologically confirmed uterine leiomyomas, it mimics key endocrine and uterine biochemical alterations associated with fibroid pathophysiology, particularly estrogen‐ and progesterone‐driven proliferative changes, thereby providing a relevant experimental platform for evaluating potential anti‐fibroid interventions.

### 3.2 Effect of *X. aethiopica* on Serum Estradiol

**FIGURE 2:**
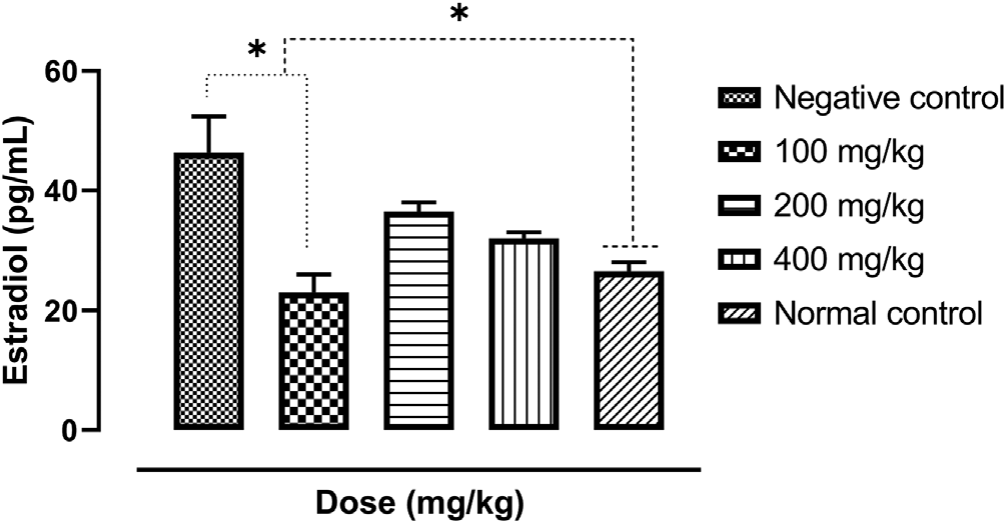
Influence of plant extract on serum estradiol level in MSG‐induced fibroid animals. The values above are mean of six replicates (*n* = 6). Values with superscripts * indicate significant difference relative value of negative control *p* ≤ 0.05.

The effect of *X. aethiopica* extract on serum estradiol concentration in MSG‐induced fibroid rats is presented in Figure 2. Administration of MSG alone (Group A) caused a significant (*p* ≤ 0.05) increase in serum estradiol level (48.56 ± 3.24 pg/mL) compared with the normal control (Group E), which recorded a mean value of 32.47 ± 2.18 pg/mL. Co‐treatment with *X. aethiopica* extract produced a marked and dose‐dependent reduction in estradiol levels relative to the MSG‐only group (Figure 2).

Specifically, the group treated with 100 mg/kg of *X. aethiopica* (Group B) exhibited a significantly lower estradiol concentration (24.35 ± 1.74 pg/mL), while the 200 and 400 mg/kg dose groups (Groups C and D) recorded values of 37.86 ± 2.01 and 35.42 ± 1.89 pg/mL, respectively. The extract‐treated groups showed no significant differences compared with the normal control group, except at the lowest dose (100 mg/kg), which produced a slightly lower value (Figure 2).

Estradiol is a key regulator of uterine growth and plays a central role in the initiation and progression of uterine fibroids by stimulating smooth muscle cell proliferation and ECM accumulation [28]. In the present study, administration of MSG alone resulted in a significant elevation of serum estradiol levels compared with the normal control group, confirming that MSG induces endocrine disruption. This finding is consistent with previous studies reporting that MSG alters HPO axis regulation through excitotoxic damage to hypothalamic neurons, leading to dysregulated gonadotropin secretion and enhanced ovarian estrogen synthesis. Sustained estrogen excess is a well‐established driver of fibroid development and uterine hypertrophy. Co‐administration of *X. aethiopica* extract significantly attenuated the MSG‐induced rise in estradiol concentration, indicating a protective and modulatory effect on estrogen homeostasis. The reduction in estradiol was generally dose‐dependent, with all extract‐treated groups showing values comparable to the normal control. The markedly lower estradiol level observed at the 100 mg/kg dose suggests a strong estrogen‐suppressive effect at low concentration, whereas the slightly higher values at 200 and 400 mg/kg may reflect a nonlinear or biphasic dose–response, a phenomenon commonly reported with phytochemical‐rich extracts due to complex interactions among bioactive constituents. The estrogen‐lowering effect of *X. aethiopica* may be attributed to its rich composition of flavonoids, alkaloids, terpenoids, and phenolic compounds, which possess potent antioxidant and anti‐inflammatory properties. These phytochemicals may protect the hypothalamus and ovaries from oxidative stress, restore normal HPO axis feedback mechanisms, and possibly inhibit aromatase activity, thereby reducing estradiol biosynthesis. Similar estrogen‐modulating effects have been reported for other antioxidant‐rich medicinal plants used in the management of reproductive disorders. Overall, the ability of *X. aethiopica* to normalize serum estradiol levels in MSG‐induced fibroid rats suggests a crucial role in counteracting estrogen‐driven fibroid pathogenesis. By restoring estrogen balance, the extract may limit uterine smooth muscle proliferation and reduce the hormonal stimulus required for fibroid growth, supporting its therapeutic potential in the management of estrogen‐dependent uterine disorders [28, 29, 30].

### 3.3 Effect of *X. aethiopica* on Bodyweight

**FIGURE 3:**
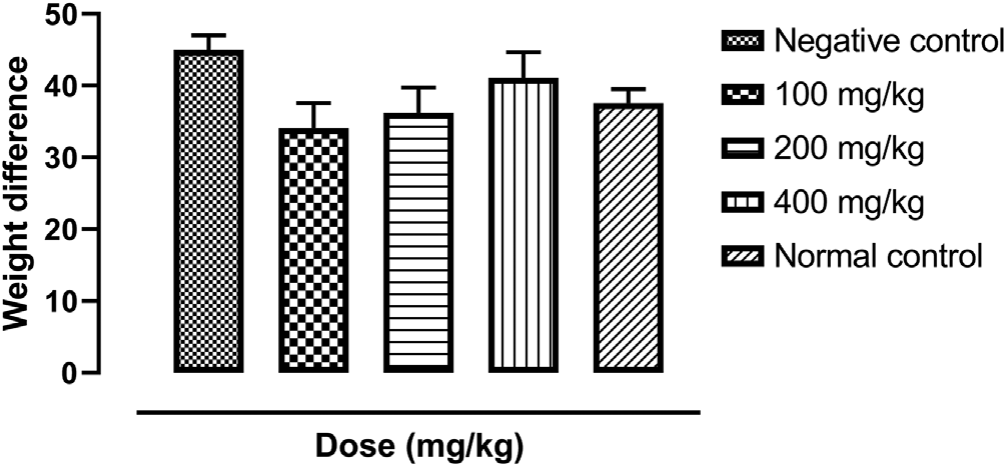
Weight difference before and after treatment episode. The values above are mean of six replicates (*n* = 6). Values with superscripts * indicate significant difference relative value of negative control *p* ≤ 0.05.

The effect of the administration of the plant extract on the body weight is shown in Figure 3 below. The changes in body weight of rats before and after treatment with *X. aethiopica* extract are presented in Figure 3. Administration of MSG alone (Group A) produced a slightly higher mean weight gain (45.36 ± 2.47 g) compared with the normal control (Group E), which recorded 38.72 ± 2.01 g. Co‐treatment with *X. aethiopica* extract produced variable but generally lower mean weight differences compared with the MSG‐only group.

Specifically, animals treated with 100 mg/kg of *X. aethiopica* (Group B) gained 33.84 ± 1.96 g, while those given 200 mg/kg (Group C) and 400 mg/kg (Group D) recorded 40.26 ± 2.17 g and 37.95 ± 1.88 g, respectively (Figure 3).

Body weight changes serve as an important indicator of general health status, metabolic integrity, and potential systemic toxicity of administered substances. In the present study, MSG‐treated rats exhibited a slightly higher mean weight gain compared with the normal control group, although this increase was not statistically significant. This trend is consistent with earlier reports suggesting that MSG may promote hyperphagia and weight gain through its action on hypothalamic neurons involved in appetite and satiety regulation. MSG‐induced alterations in insulin and leptin signaling have also been implicated in increased feed intake and adiposity in experimental animals [31]. However, the absence of a significant difference in this study indicates that the dose and duration of MSG exposure were insufficient to induce overt metabolic dysfunction. Co‐administration of *X. aethiopica* extract resulted in variable but generally lower weight gain relative to the MSG‐only group, with the 100 mg/kg dose producing the lowest mean weight increase. Importantly, none of the extract‐treated groups differed significantly from the normal control, suggesting that the extract did not impair growth or nutritional status. This finding indicates that *X. aethiopica* is well tolerated at the administered doses and does not exert systemic toxicity that could manifest as weight loss or growth retardation. The slight moderation of weight gain observed in the extract‐treated groups may reflect improved metabolic regulation and reduced oxidative stress, consistent with previous studies reporting antioxidant, hypolipidemic, and hepatoprotective properties of *X. aethiopica*. By preserving metabolic homeostasis, the extract may counteract subtle MSG‐induced metabolic effects without disrupting normal physiological growth. Overall, the body weight data support the safety profile of *X. aethiopica* and indicate that its beneficial effects on reproductive hormones occur independently of adverse systemic or metabolic effects. This further strengthens its potential suitability for long‐term use in the management of MSG‐induced reproductive and uterine disorders [31, 32].

**FIGURE 4:**
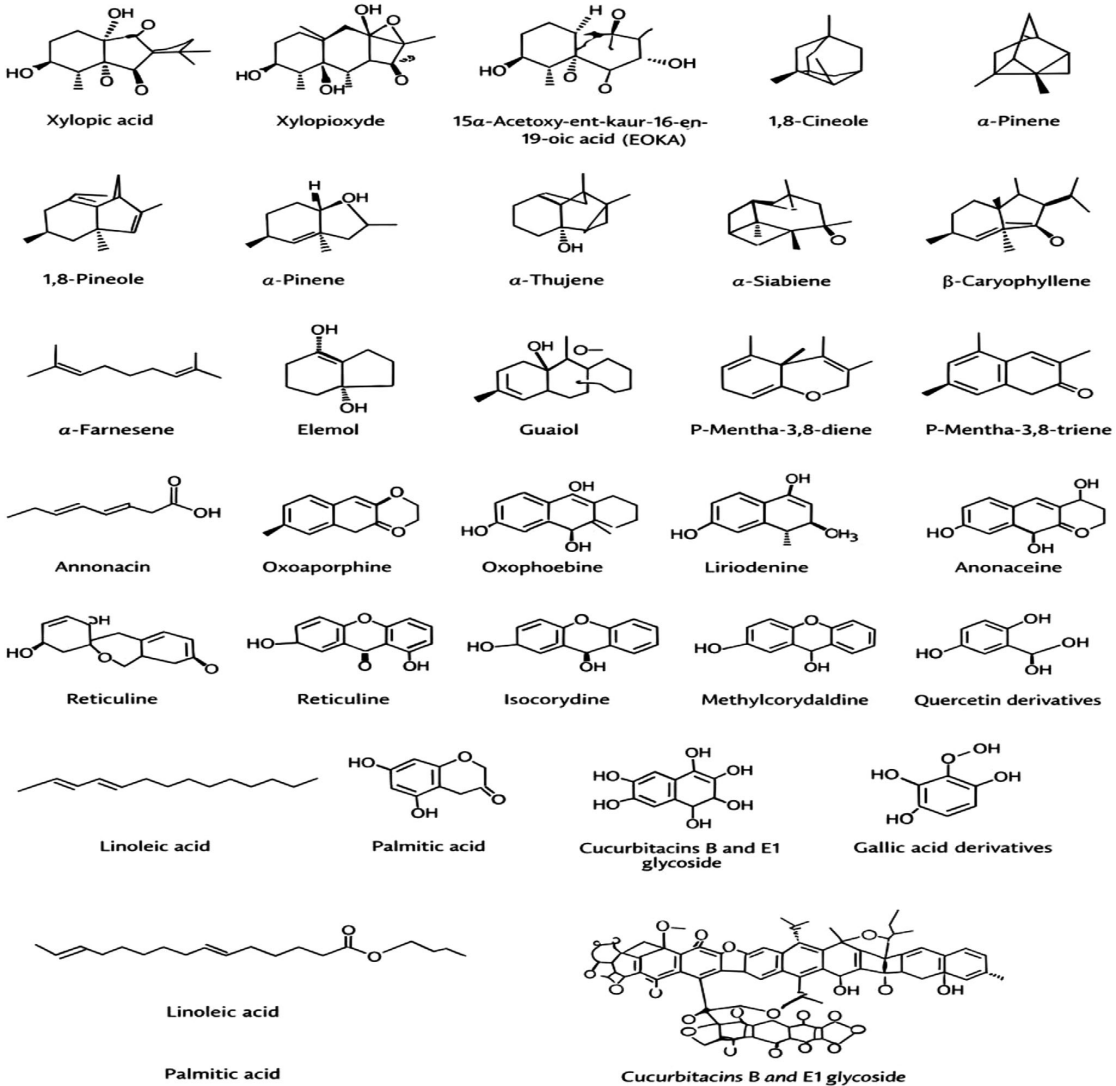
Review of compounds isolated from *Xylopia aethiopica*.

Figure 4 presents a comprehensive overview of the major phytochemical constituents reported to be isolated from *X. aethiopica* [33]. The compounds span several structural classes, reflecting the chemical diversity of the plant. These include diterpenoids such as xylopic acid, xylopiosyde, and 15α‐acetoxy‐ent‐kaur‐16‐en‐19‐oic acid, as well as monoterpenes and sesquiterpenes including 1,8‐cineole, α‐pinene, α‐thujene, α‐sabinene, β‐caryophyllene, α‐farnesene, p‐mentha‐3,8‐diene, and p‐mentha‐3,8‐triene [33]. Oxygenated terpenoids such as elemol and guaiol are also represented. In addition, several alkaloids characteristic of the Annonaceae family are shown, including oxoaporphine, oxophoebine, liriodenine, anonaine, reticuline, isocorydine, and methylcorydaldine. The figure further highlights the presence of phenolic and polyphenolic compounds, notably quercetin derivatives and gallic acid derivatives, alongside fatty acids such as linoleic acid and palmitic acid. High‐molecular‐weight bioactive constituents, including cucurbitacins B and E1 glycosides, are also illustrated. Collectively, the diversity of compounds depicted in this figure underscores the rich phytochemical profile of *X. aethiopica* and provides a chemical basis for its wide range of reported pharmacological activities (Figure 4) [33].

It is noteworthy to state that the limitation of the present study is the absence of histopathological and molecular confirmation of uterine fibroid lesions. Consequently, the observed hormonal and uterine changes should be interpreted as reflecting fibroid‐associated pathophysiological alterations rather than definitive evidence of fibroid formation. Future studies incorporating histological examination, ECM assessment, and fibroid‐specific biomarkers are required to further validate the model.

### 3.4 Molecular Mechanism Result

Uterine fibroids (leiomyomas) are benign monoclonal tumors of uterine smooth muscle cells characterized by excessive ECM deposition, increased cellular proliferation, and altered tissue remodeling. Their pathogenesis is multifactorial and involves genetic susceptibility, particularly mutations such as MED12, hormonal dysregulation, chronic inflammation, and aberrant growth factor signaling. Estrogen and progesterone are central drivers of fibroid development, where estrogen primarily enhances the expression of progesterone receptors and promotes cellular proliferation, while progesterone stimulates tumor growth by increasing ECM production and inhibiting apoptosis. These hormonal effects are further amplified through downstream signaling pathways involving transforming growth factor‐β (TGF‐β), vascular endothelial growth factor (VEGF), epidermal growth factor (EGF), and platelet‐derived growth factor (PDGF), which collectively contribute to fibrosis, angiogenesis, and sustained tumor expansion [34, 35]. In recent years, phytotherapeutic interventions have gained increasing attention as alternative or complementary strategies for uterine fibroid management due to the limitations of conventional hormonal and surgical treatments. Medicinal plants exert anti‐fibroid effects through multiple mechanisms, including modulation of steroid hormone receptors, inhibition of smooth muscle cell proliferation, induction of apoptosis, and suppression of inflammatory and fibrotic signaling pathways. Bioactive phytochemicals such as flavonoids, alkaloids, and polyphenols have been shown to interfere with estrogen and progesterone signaling as well as downregulate TGF‐β‐mediated ECM deposition, thereby reducing fibroid growth and progression [36, 37]. These multi‐target actions support the growing interest in plant‐based compounds as potential therapeutic agents in hormone‐dependent reproductive disorders.

At the molecular level, uterine fibroid progression is driven by complex interactions between steroid hormone receptors, growth factor signaling pathways, and ECM remodeling networks. Progesterone receptor activation plays a key role in promoting fibroid cell survival and proliferation, while estrogen signaling enhances tissue responsiveness to progesterone and contributes to sustained tumor growth. In addition, TGF‐β signaling is a major regulator of fibrosis through stimulation of collagen synthesis and ECM accumulation, whereas VEGF‐mediated angiogenesis ensures adequate vascular support for expanding fibroid tissue. Collectively, these pathways form an interconnected regulatory network that underpins fibroid development and progression. Consequently, targeting multiple nodes within these pathways has emerged as a promising therapeutic strategy, particularly through multi‐target agents such as phytochemicals capable of simultaneously modulating hormonal, angiogenic, and fibrotic signaling cascades [38, 39, 40].

The molecular docking analysis of compounds isolated from *X. aethiopica* revealed promising interactions with multiple protein targets implicated in fibroid pathophysiology (Table 1). Docking scores were compared with those of native ligands to assess the relative binding potential of the phytochemicals.

**TABLE 1:** Molecular docking binding energies of major *Xylopia aethiopica*‐derived compounds against selected protein targets.

Protein (PDB ID)	Ligand	Binding energy (kcal/mol)
Progesterone receptor (2W8Y)	Mifepristone (native)	−9.4
	Xylopioxyde	−9.6
	Liriodenine	−9.4
VEGFR2 receptor (3VHE)	42q (native)	−10.2
	Tiriloside	−10.0
	Xylopioxyde	−9.0
	Liriodenine	−8.6
TGF‐beta receptor type 1 kinase domain (5E9O)	Native ligand	−9.7
	Liriodenine	−10.4
	Aporphine	−9.8
	Oxophoebine	−9.8
Human estrogen receptor beta receptor (1L2J)	ETC (native)	−11.4
	Xylopioxyde	−9.7
	Liriodenine	−9.6
5GS4	EST (NATIVE LIGAND)	−10
	LIRIODENINE	−9.6
	APORPHINE	−9.0

For the progesterone receptor (2W8Y), xylopioxyde exhibited the highest binding affinity (−9.6 kcal/mol), slightly surpassing the native ligand mifepristone (−9.4 kcal/mol). This indicates that xylopioxyde may effectively interact with the oxytocin receptor, potentially modulating receptor activity and contributing to uterotonic effects. Liriodenine also showed comparable affinity (−9.4 kcal/mol), suggesting that multiple compounds from *X. aethiopica* could target the oxytocin receptor (Table 1).

Docking against VEGFR2 receptor (3VHE) showed that tiriloside achieved a strong binding energy (−10.0 kcal/mol), closely approaching the native ligand native ligand co‐crystallized with VEGFR2 (3VHE) (42Q; −10.2 kcal/mol). Xylopioxyde (−9.0 kcal/mol) and liriodenine (−8.6 kcal/mol) displayed moderate affinities (Table 1).

For TGF‐beta receptor type 1 kinase domain (5E9O), liriodenine exhibited the strongest binding (−10.4 kcal/mol), exceeding the native ligand (−9.7 kcal/mol) (Table 1). Both alkaloid compounds isolated from *X. aethiopica* (aporphine) and oxophoebine also displayed moderate affinities (−9.8 kcal/mol) (Table 1). In the human estrogen receptor beta receptor (1L2J), xylopioxyde and liriodenine demonstrated significant binding (−9.7 and −9.6 kcal/mol, respectively), although lower than the native ligand ETC (−11.4 kcal/mol) (Table 1). Finally, for ESR1 (5GS4), which is also implicated in uterine contractility pathways, the native ligand showed the strongest binding (−10.0 kcal/mol), while liriodenine (−9.6 kcal/mol) and aporphine (−9.0 kcal/mol) demonstrated moderate binding. This reinforces the potential multi‐target activity of *X. aethiopica* compounds support the potential multi‐target pharmacological activity of *X. aethiopica* alkaloids (Table 1).

**FIGURE 5:**
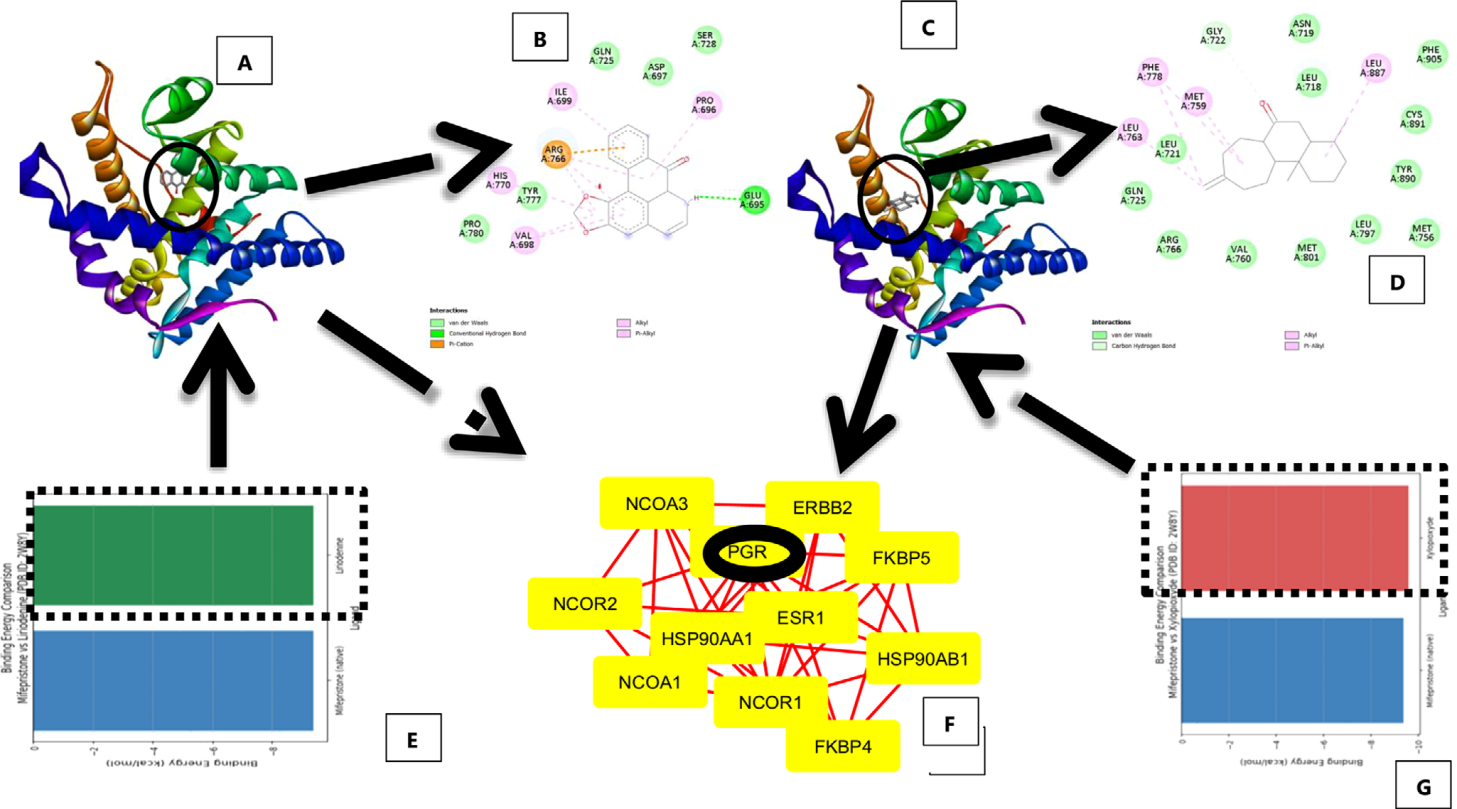
Illustrates the binding interactions, comparative binding energies, and protein–protein interaction (PPI) network associated with the progesterone receptor (PGR), highlighting the mechanistic basis of *Xylopia aethiopica* activity in uterine fibroid modulation. (A) Three‐dimensional (3D) ribbon representation of the progesterone receptor (PGR; PDB ID: 2W8Y) complexed with liriodenine, showing the ligand occupying the receptor's ligand‐binding domain. The protein structure is displayed as a multicolored ribbon, while the ligand is shown in stick representation, indicating stable accommodation within the active pocket. (B) Two‐dimensional (2D) interaction map of liriodenine within the PGR binding site. The ligand forms multiple stabilizing interactions, including van der Waals forces, π–alkyl interactions, and hydrogen bonding with key residues such as ARG766, GLU695, PRO696, VAL698, and ILE699, which are critical for progesterone receptor activation and signaling. (C) 3D ribbon representation of PGR in complex with xylopioxyde, another bioactive diterpene from *Xylopia aethiopica*, showing a binding orientation comparable to that of known progesterone receptor antagonists. (D) 2D interaction diagram of xylopioxyde–PGR complex, highlighting hydrophobic and π–alkyl interactions with residues including LEU718, LEU721, MET759, PHE778, and ARG766, supporting strong ligand stabilization within the receptor pocket. (E) Comparative binding energy analysis of liriodenine and the standard progesterone receptor antagonist mifepristone. Liriodenine demonstrates a binding affinity comparable to mifepristone, supporting its potential antagonistic activity against PGR. (F) STRING‐based protein–protein interaction (PPI) network of the progesterone receptor (PGR). PGR is shown as a central hub interacting with key regulatory proteins, including estrogen receptor alpha (ESR1), nuclear receptor co‐activators (NCOA1, NCOA3), co‐repressors (NCOR1, NCOR2), molecular chaperones (HSP90AA1, HSP90AB1), immunophilins (FKBP4, FKBP5), and growth signaling receptor ERBB2. These interactions underline extensive crosstalk between progesterone signaling, estrogen signaling, and proliferative pathways. (G) Comparative binding energy analysis of xylopioxyde vs. mifepristone, showing that xylopioxyde exhibits binding energy equal to or slightly stronger than the native antagonist, reinforcing its potential role as a natural progesterone receptor modulator.

**FIGURE 6:**
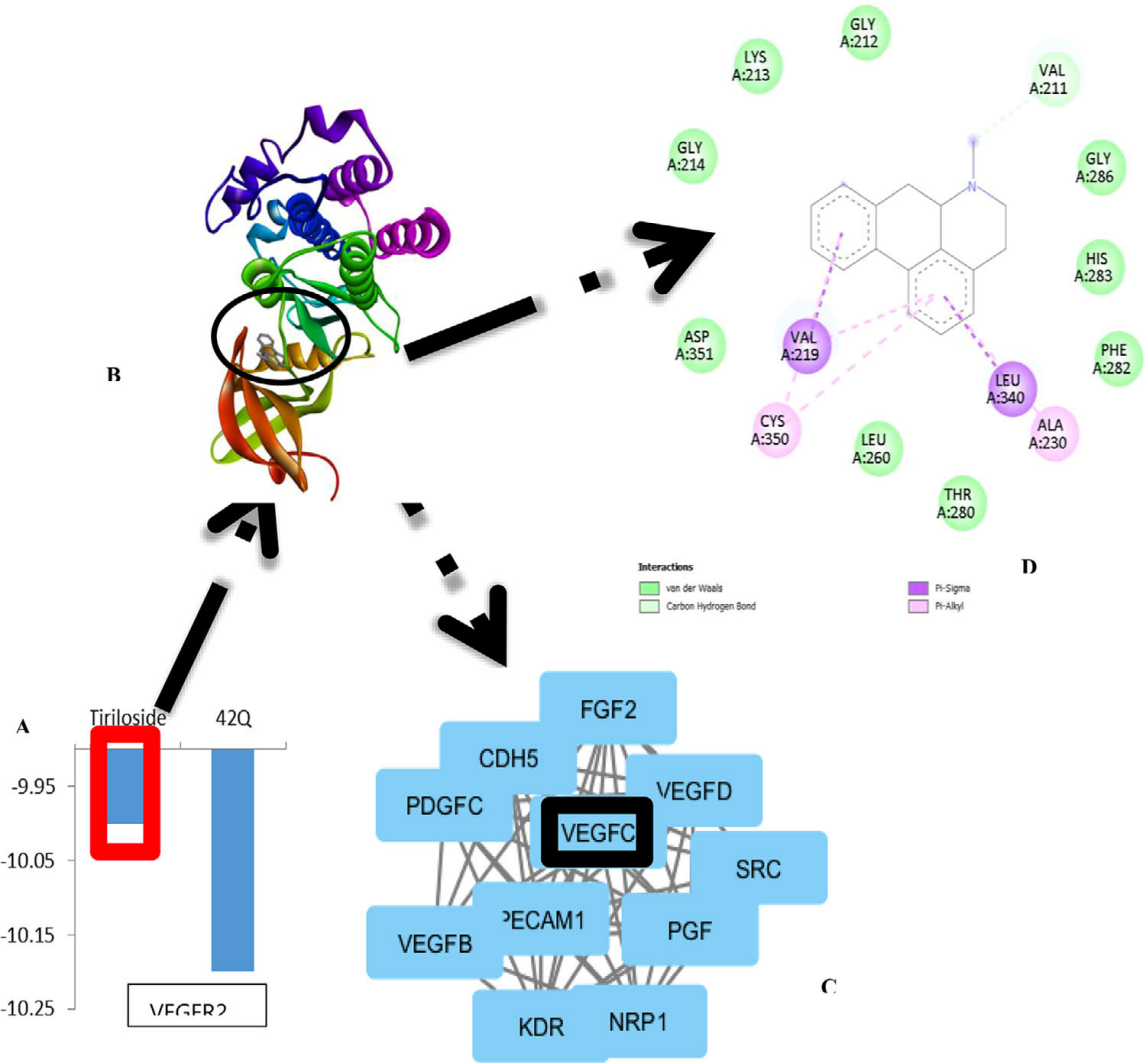
Molecular docking and protein–protein interaction (PPI) analyses illustrating the anti‐angiogenic potential of tiriloside, a bioactive compound from *Xylopia aethiopica*, through targeting of the vascular endothelial growth factor receptor 2 (VEGFR2; PDB ID: 3VHE). (A) Comparative binding affinity of tiriloside and the native co‐crystallized ligand 42Q, showing a binding energy of −10.0 kcal/mol for tiriloside, comparable to that of 42Q (−10.2 kcal/mol). (B) Three‐dimensional (3D) ribbon representation of the VEGFR2–tiriloside complex demonstrating stable accommodation of tiriloside within the receptor's ligand‐binding pocket and favorable steric complementarity with the active site. (D) Two‐dimensional (2D) interaction map revealing that tiriloside is stabilized predominantly by hydrophobic interactions, including π–alkyl and π–sigma contacts with key residues such as VAL219, LEU430, CYS350, and ALA230, in addition to van der Waals interactions with surrounding residues including GLY212, LYS213, HIS283, and PHE282. These residues are located within functional regions critical for VEGFR2 activation and downstream kinase signaling, suggesting that tiriloside may interfere with VEGFR2‐mediated signal transduction. (C) STRING‐based PPI network centered on vascular endothelial growth factor C (VEGFC), highlighting interactions with major angiogenic and proliferative regulators, including VEGFB, VEGFD, PDGFC, FGF2, PGF, SRC, KDR (VEGFR2), NRP1, CDH5, and PECAM1. The dense connectivity within this network underscores the central role of the VEGFC–VEGFR2 signaling axis in angiogenesis and lymphangiogenesis, processes essential for vascular development and sustained growth of uterine fibroids.

Figure 5 provides integrated molecular docking, binding energy comparison, and protein–protein interaction (PPI) analyses that collectively elucidate the mechanistic basis through which *X. aethiopica* bioactive compounds may modulate progesterone receptor (PGR) signaling in uterine fibroids. The 3D docking representations (Figure 6A,C) demonstrate that both liriodenine and xylopioxyde stably occupy the ligand‐binding domain of PGR, adopting conformations comparable to those of established progesterone receptor antagonists. This suggests favorable steric and energetic compatibility with the receptor's active pocket. Although molecular docking provided useful insights into potential ligand–target interactions, these results require further validation through in vitro and in vivo experimental studies.

The 2D interaction analyses (Figure 6B,D) further reveal that both ligands engage key amino acid residues critical for PGR activation and downstream signaling. Liriodenine forms hydrogen bonding and π–alkyl interactions with residues such as ARG766, GLU695, PRO696, VAL698, and ILE699, which are known to contribute to ligand stabilization and receptor conformational changes. Similarly, xylopioxyde predominantly interacts through hydrophobic and π–alkyl contacts involving LEU718, LEU721, MET759, PHE778, and ARG766, indicating a strong affinity for the hydrophobic core of the ligand‐binding pocket. These interaction patterns suggest that the compounds may interfere with progesterone‐induced receptor activation. Comparative binding energy analyses (Figure 6E,G) show that both liriodenine and xylopioxyde exhibit binding affinities comparable to, or slightly stronger than, mifepristone, a clinically used progesterone receptor antagonist. This finding supports the possibility that these plant‐derived compounds may function as natural PGR modulators, capable of attenuating progesterone‐driven cellular proliferation associated with fibroid growth. The STRING‐based PPI network (Figure 5F) positions PGR as a central regulatory hub interacting with ESR1, nuclear receptor co‐activators (NCOA1, NCOA3), co‐repressors (NCOR1, NCOR2), molecular chaperones (HSP90AA1, HSP90AB1), immunophilins (FKBP4, FKBP5), and ERBB2. This extensive interaction network highlights the crosstalk between progesterone signaling, estrogen signaling, and growth factor‐mediated proliferative pathways, all of which are implicated in uterine fibroid pathophysiology [41]. Modulation of PGR activity by *X. aethiopica* compounds may therefore exert broader regulatory effects beyond progesterone signaling alone. Overall, the findings from Figure 5 provide strong in silico evidence that liriodenine and xylopioxyde can interact effectively with the progesterone receptor and potentially disrupt hormone‐driven signaling cascades involved in fibroid development. These results complement the observed in vivo hormonal modulation and support the therapeutic potential of *X. aethiopica* as a natural anti‐fibroid agent. However, further experimental validation is required to confirm these molecular interactions and their biological consequence [41, 42].

**FIGURE 7:**
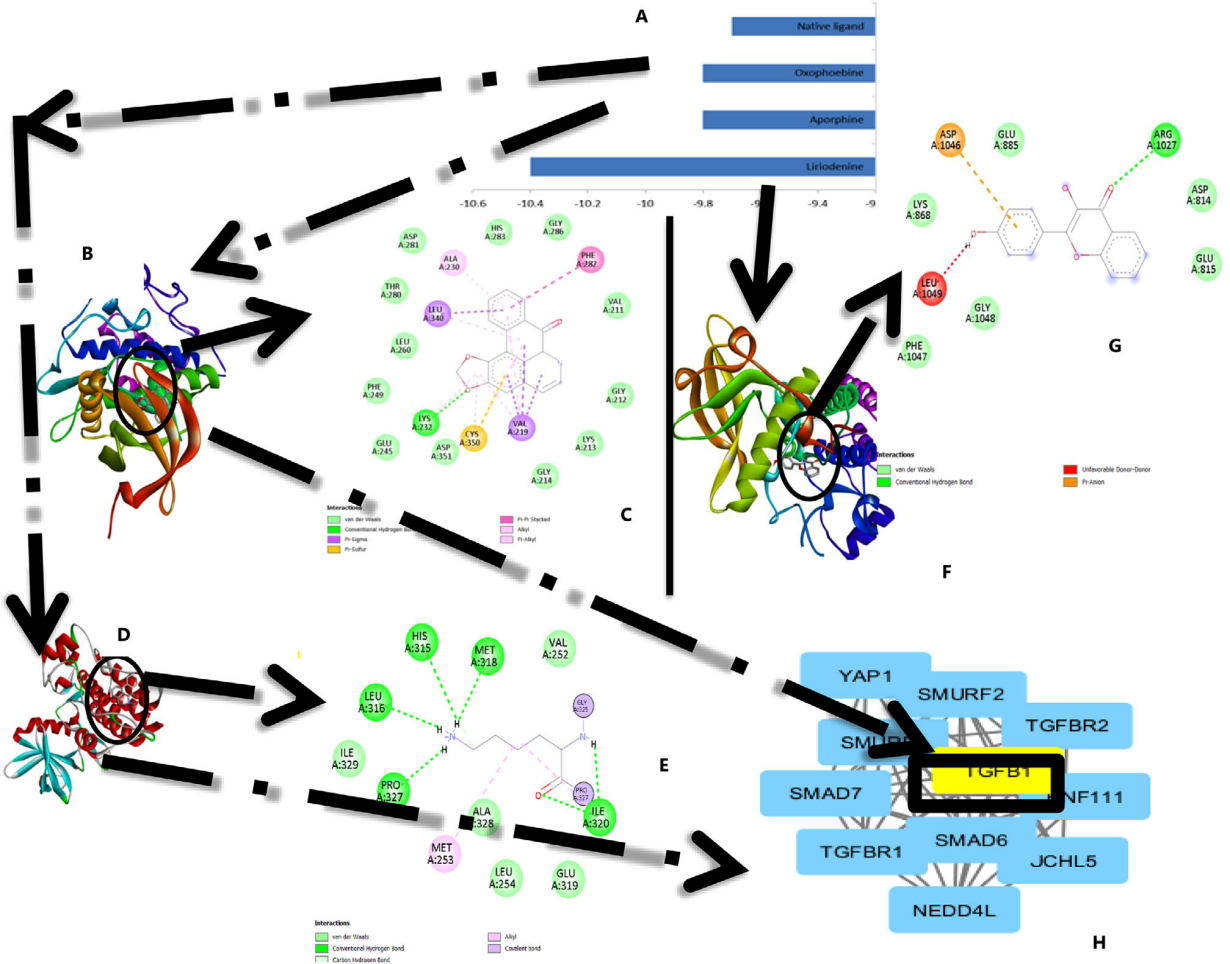
Illustrates the binding interactions, comparative binding energies, and protein–protein interaction (PPI) network associated with the TGF‐beta1 highlighting the mechanistic basis of *Xylopia aethiopica* activity in uterine fibroid modulation.

Angiogenesis plays a critical role in the pathophysiology of uterine fibroids by supporting sustained tissue growth through enhanced vascularization and nutrient supply [5, 19, 20]. In this study, molecular docking analysis revealed that tiriloside, a bioactive compound from *X. aethiopica*, binds strongly to the vascular endothelial growth factor receptor 2 (VEGFR2; PDB ID: 3VHE) Figure 6A, a key regulator of angiogenic signaling. Tiriloside exhibited a binding affinity of −10.0 kcal/mol Figure 6A, which is comparable to that of the native co‐crystallized ligand, 42Q (−10.2 kcal/mol), suggesting a high likelihood of effective receptor engagement and competitive inhibition at the VEGFR2 active site Figure 6A. The close similarity in binding energies indicates that tiriloside may possess sufficient structural complementarity to occupy the ligand‐binding pocket of VEGFR2 and potentially disrupt receptor activation Figure 7B,D.

Structural interaction analysis further demonstrated that tiriloside is stabilized within the VEGFR2 binding pocket predominantly through hydrophobic interactions, including π–alkyl and π–sigma contacts with key residues such as VAL219, LEU430, CYS350, and ALA230, along with extensive van der Waals interactions involving GLY212, LYS213, HIS283, and PHE282 Figure 6D. Many of these residues are located within functional regions essential for kinase activity and downstream signal transduction, implying that tiriloside binding could interfere with VEGFR2 autophosphorylation and subsequent activation of angiogenic signaling cascades Figure 6D. Hydrophobic interactions of this nature are known to contribute significantly to ligand stability and residence time within receptor active sites, thereby enhancing inhibitory potential Figure 6B. In addition to direct receptor targeting, the STRING‐based PPI network analysis highlighted the central role of VEGFC in angiogenesis and lymphangiogenesis (Figure 6C), revealing extensive interactions with key angiogenic regulators, including VEGFB, VEGFD, PDGFC, FGF2, PGF, SRC, KDR (VEGFR2), NRP1, CDH5, and PECAM1 Figure 6C. The dense connectivity within this network underscores the importance of VEGFC–VEGFR2 signaling in vascular development and pathological tissue proliferation. Disruption of this axis has been widely implicated as an effective strategy for suppressing aberrant angiogenesis in fibroid growth. Therefore, inhibition of VEGFR2 by tiriloside may exert downstream effects on VEGFC‐mediated signaling, amplifying its anti‐angiogenic impact (Figure 6C). Collectively, these findings suggest that tiriloside may exert anti‐fibroid effects through a multi‐target mechanism involving both hormonal modulation, as previously demonstrated through progesterone receptor interactions, and suppression of angiogenic pathways mediated by VEGFR2 and VEGFC (Figure 6C). By limiting angiogenic signaling, tiriloside may reduce blood vessel formation and nutrient delivery to fibroid tissue, thereby restricting fibroid growth and progression. While the in silico results provide compelling mechanistic insights, further in vitro and in vivo studies are required to validate the inhibitory effects of tiriloside on VEGFR2 signaling (Figure 6C) and to confirm its therapeutic potential in uterine fibroid management [43, 44, 45].

Figure 7 elucidates the anti‐fibrotic mechanism of selected alkaloid constituents of *X. aethiopica* through molecular docking, binding energy comparison, and PPI network analysis targeting the transforming growth factor‐beta (TGF‐β) signaling pathway, a central driver of uterine fibroid development and ECM accumulation. Figure 7A presents the binding affinities of three alkaloid compounds—liriodenine, aporphine, and oxophoebine—docked against the TGF‐β receptor type 1 kinase domain (TGFBR1; PDB ID: 5E9O) and compared with the native ligand. Liriodenine exhibited the highest binding affinity (−10.4 kcal/mol), surpassing that of the native ligand (−9.7 kcal/mol), while both aporphine and oxophoebine demonstrated comparable affinities (−9.8 kcal/mol). These results indicate strong thermodynamic favorability and suggest that the alkaloids, particularly liriodenine, may act as potent inhibitors of TGFBR1 kinase activity. Figure 7B (3D ribbon structure) and 7C (2D interaction map) illustrate the binding mode of liriodenine within the active site of TGFBR1. The ligand is stably accommodated within the kinase domain, exhibiting favorable steric complementarity. The 2D interaction map reveals that liriodenine is stabilized through a combination of conventional hydrogen bonds, π–alkyl interactions, π–sigma interactions, and van der Waals forces with key residues including VAL219, LEU430, CYS350, ALA230, GLY212, and LYS213. These residues are located within functionally important regions of the kinase domain, implying that liriodenine may effectively block ATP binding or substrate phosphorylation, thereby suppressing downstream fibrotic signaling.

Figure 7D (3D ribbon) and 7E (2D interaction diagram) depict the interaction of oxophoebine with TGFBR1. Oxophoebine occupies a similar binding pocket as liriodenine, forming stabilizing hydrogen bonds and hydrophobic interactions with residues such as LEU, MET, GLU, HIS, and VAL. Although its binding affinity (−9.8 kcal/mol) is slightly lower than that of liriodenine, the interaction profile suggests effective receptor engagement and potential inhibition of kinase activation. This supports oxophoebine's role as a complementary anti‐fibrotic agent within the phytochemical matrix of *X. aethiopica*.

Figure 7F (3D ribbon) and 7G (2D interaction map) show the binding conformation of aporphine within the TGFBR1 kinase domain. Aporphine establishes multiple π–alkyl and van der Waals interactions, contributing to its stable positioning in the active site. The observed binding affinity (−9.8 kcal/mol) comparable to oxophoebine and superior to the native ligand indicates that aporphine may also disrupt TGF‐β receptor signaling. Collectively, these docking results demonstrate that all three alkaloids possess favorable binding characteristics against TGFBR1, with liriodenine emerging as the most potent.

Figure 7H illustrates the STRING‐derived PPI network centered on TGF‐β1 (TGFB1), highlighting its extensive interactions with TGFBR1, TGFBR2, SMAD2/3, SMAD6, SMAD7, SMURF2, YAP1, and NEDD4L. TGFB1 acts as a master regulator of fibrotic signaling, driving ECM deposition, collagen synthesis, and smooth muscle cell proliferation—hallmark features of uterine fibroids. The dense connectivity of this network underscores the complexity of TGF‐β signaling and emphasizes that inhibition of TGFBR1 can disrupt multiple downstream fibrogenic pathways simultaneously [46, 47].

It is important to note that the present study employed a crude methanolic extract of *X. aethiopica*, and therefore the specific bioactive compounds responsible for the observed hormonal and uterine effects were not isolated or experimentally validated. The molecular docking analysis was performed using phytochemicals previously reported in the literature for *X. aethiopica* and was intended to provide preliminary, hypothesis‐generating insight into possible molecular interactions rather than to establish direct causality. Furthermore, the selection of molecular targets was based on established literature on uterine fibroid pathophysiology and was not derived from transcriptomic or disease‐model‐specific gene expression profiling. Consequently, the in silico findings should be interpreted as supportive and exploratory evidence of potential multi‐target mechanisms rather than definitive mechanistic proof. Future studies incorporating compound isolation, quantitative phytochemical profiling, transcriptomic or proteomic analyses, and experimental validation of predicted targets are warranted to further elucidate the precise molecular mechanisms underlying the anti‐fibroid effects of *X. aethiopica*.

## 4 Conclusions

This study provides comprehensive in vivo and in silico evidence supporting the therapeutic potential of *X. aethiopica* in the management of uterine fibroids through multi‐mechanistic modulation of hormonal, angiogenic, and fibrotic pathways. In the MSG‐induced fibroid rat model, *X. aethiopica* extract significantly normalized elevated serum progesterone and estradiol levels without adversely affecting body weight or general physiological status, indicating effective endocrine regulation alongside a favorable safety profile. Molecular docking analyses further elucidated the mechanistic basis of these effects, demonstrating that key phytoconstituents—including liriodenine, xylopioxyde, aporphine, and tiriloside—exhibited strong and stable binding interactions with multiple molecular targets implicated in fibroid pathogenesis. Notably, xylopioxyde and liriodenine showed binding affinities comparable to or exceeding that of the standard antagonist mifepristone at the progesterone receptor, supporting their potential role as natural progesterone receptor modulators. Tiriloside displayed high affinity toward VEGFR2, suggesting an additional anti‐angiogenic mechanism, while interactions with TGF‐β receptor type 1 point to possible attenuation of fibrotic signaling. PPI network analyses revealed extensive crosstalk between progesterone signaling, estrogen signaling, angiogenesis, and growth factor–mediated proliferative pathways, positioning these receptors as central hubs in fibroid biology. The ability of *X. aethiopica* constituents to target multiple nodes within these interconnected networks underscores a pleiotropic pharmacological profile, which may offer advantages over conventional single‐target therapies. Collectively, the findings demonstrate that *X. aethiopica* exerts coordinated hormonal, anti‐angiogenic, and anti‐fibrotic effects that are relevant to uterine fibroid progression. This multi‐target activity supports its ethnomedicinal use and highlights its promise as a natural, non‐surgical, and fertility‐sparing therapeutic candidate for uterine fibroids. Although, our findings suggested that *X. aethiopica* may modulate hormonal and uterine alterations associated with uterine fibroid pathophysiology in the experimental model employed. However, further studies incorporating histopathological and molecular validation are necessary to confirm its therapeutic potential against uterine fibroids, also further studies involving compound isolation, pharmacokinetic evaluation, and targeted in vitro and in vivo validation are warranted to translate these findings into clinical application.

## Author Contributions

Adedokun Oluwasegun: conceptualization, writing – original draft, methodology, formal analysis, supervision, and resources. Ume Ogochukwu: conceptualization, writing – original draft, methodology, formal analysis, supervision, and resources. Akinsola Olubiyi: formal analysis, methodology, writing, resources, and review and editing. Agada Emmanuel: formal analysis, methodology, writing, resources, and review and editing. Adebiyi Omowumi: formal analysis, methodology, writing, resources, and review and editing. Idogho Eseoghena: formal analysis, methodology, writing, resources, and review and editing. Adedokun Folake: formal analysis, methodology, writing, resources, and review and editing. Adekunle Temilade: formal analysis, methodology, writing, resources, and review and editing.

## Conflicts of Interest

The authors declare no conflicts of interest.

## Data Availability

The data that support the findings of this study are available from the corresponding author upon reasonable request.
